# Polo-Like Kinase 3 Appears Dispensable for Normal Retinal Development Despite Robust Embryonic Expression

**DOI:** 10.1371/journal.pone.0150878

**Published:** 2016-03-07

**Authors:** Jillian J. Goetz, Lauren A. Laboissonniere, Andrea K. Wester, Madison R. Lynch, Jeffrey M. Trimarchi

**Affiliations:** 1 Department of Genetics, Development and Cell Biology, Iowa State University, Ames, Iowa, United States of America; 2 Ames High School, Ames, Iowa, United States of America; Universidade Federal do ABC, BRAZIL

## Abstract

During retinogenesis seven different cell types are generated in distinct yet overlapping timepoints from a population of retinal progenitor cells. Previously, we performed single cell transcriptome analyses of retinal progenitor cells to identify candidate genes that may play roles in the generation of early-born retinal neurons. Based on its expression pattern in subsets of early retinal cells, polo-like kinase 3 (Plk3) was identified as one such candidate gene. Further characterization of Plk3 expression by *in situ* hybridization revealed that this gene is expressed as cells exit the cell cycle. We obtained a Plk3 deficient mouse and investigated changes in the retina’s morphology and transcriptome through immunohistochemistry, *in situ* hybridization and gene expression profiling. These experiments have been performed initially on adult mice and subsequently extended throughout retinal development. Although morphological studies revealed no consistent changes in retinogenesis upon Plk3 loss, microarray profiling revealed potential candidate genes altered in Plk3-KO mice. Further studies will be necessary to understand the connection between these changes in gene expression and the loss of a protein kinase such as Plk3.

## Introduction

Neural progenitor cells acquire their cell fates at particular times and places to ensure the right connections are formed in concert to generate an intricately functioning and responsive tissue. Although numerous environmental and cell-intrinsic factors have been shown to contribute to the fate decisions of neural progenitor cells, there are many players still to be identified [1]. Importantly, single-cell transcriptomic analyses have shown that cycling retinal progenitor cells exhibit extreme diversity in their expression of known transcription factors and markers of various cell processes [2]. However, the function of many of the dynamically expressed genes has not been studied and, therefore, the phenotypic repercussions of this gene expression heterogeneity are not understood. A better understanding of the effects on cell fate acquisition and retinal cell differentiation through gain and loss of function of these genes will greatly inform our knowledge of how a complex neural tissue is generated.

Central nervous system development can be modeled using the vertebrate retina due to its relative simplicity, laminar organization, and ease of isolation. The retina’s six major neural types are organized into three nuclear layers with rod and cone photoreceptors in the outer nuclear layer (ONL); horizontal, bipolar, and amacrine interneurons in the inner nuclear layer (INL); and retinal ganglion cells (RGCs) and displaced amacrine cells in the ganglion cell layer (GCL) [3]. The developmental timeline during which these cells are generated has been well-characterized and is common to all vertebrates, beginning with ganglion cells and other early-born cell types such as cone photoreceptors, horizontal cells, and amacrine cells and ending with the production of rod photoreceptors, bipolar cells, and Müller glia [4–7]. Clonal analyses have determined that retinal progenitors are multipotent, or capable of producing more than one type of retinal cell throughout development [8, 9]. While the exact mechanism by which dividing retinal progenitor cells assume a cell fate is not fully elucidated, studies of individual retinal progenitor cells did reveal considerable gene expression heterogeneity throughout the different stages of retinogenesis and identified many new genes with expression patterns that correlated with the generation of different retinal cells [2].

Math5 is a bHLH transcription factor expressed in retinal progenitor cells late in the cell cycle, when cell fates are most likely being acquired [10, 11]. In the mouse, a subset of early-generated retinal progenitor cells, including photoreceptors, amacrine cells, horizontal cells, and a majority of ganglion cells show a history of Math5 expression [10, 12–14]. Furthermore, Math5 and its homologues are necessary for ganglion cell generation and optic nerve formation in multiple vertebrates, including zebrafish and mice [15–17]. In addition, Math5 deficiency leads to altered proportions of other early retinal cells, indicating that this transcription factor is important in early retinal cell development [12, 13, 15, 16, 18, 19]. Given the critical and conserved expression of Math5 in early retinogenesis, we identified genes highly correlated with Math5 expression in the transcriptomes of single retinal progenitors and developing neurons isolated throughout retinogenesis (Trimarchi & Cepko, *in preparation*). Among the genes most highly correlated with Math5 expression in single retinal cells was Polo-like kinase 3 (Plk3).

The vertebrate polo-like kinases, much like their *Drosophila* homologue, Polo, can behave as key cell cycle regulators [20–22]. In mammalian cells, Polo-like kinase 1 (Plk1) plays the canonical role of controlling entry into M phase, whereas the precise roles of Plk2 and Plk3 are less clear [21]. While Plk3 expression has been detected throughout the cell cycle in cultured cells, peak Plk3 protein expression has been found to occur during the G1 phase of the cell cycle. Downregulation of Plk3 in these same cultured cells showed that this kinase is a key regulator of the G1 to S phase transition through post-transcriptional attenuation of Cyclin E, possibly in conjunction with its substrate, Cdc25A [20, 22, 23]. Additionally, Plk3 has been linked to the p53 pathway, possibly playing a role in cell cycle arrest and apoptosis [24]. Other studies, however, have pointed to possible roles for Plk2 and Plk3 outside of the cell cycle. For example, Plk2 and Plk3 have been linked to the integrity of hippocampal neurites and synaptic plasticity [25, 26]. Plk3 has also been shown to phosphorylate alpha- and beta-synuclein and along with other Plk family members it is often co-localized with phosphorylated synucleins [27]. Despite these glimpses into Plk3 function, there is no study that examines its specific role during the development of the retina.

To ascertain the function of Plk3 in retinal development we first characterized its expression, both in single retinal cells and on retinal sections. Contrary to previous findings in cultured cells, Plk3 is expressed primarily in newly postmitotic retinal cells throughout the early stages of retinogenesis, with its expression becoming undetectable by the time the animal is born. Furthermore, we obtained a Plk3-knockout (KO) mouse [23] to examine both the development of the retina and the mature retina in the absence of Plk3. However, extensive morphological analyses of mature and developing Plk3-KO mice revealed that loss of Plk3 does not lead to significant disruptions to the mature retinal architecture. To probe deeper, transcriptomic analyses were utilized to reveal significant changes in the expression levels of multiple gene markers. These changes were further confirmed by real-time quantitative PCR (qPCR), but to our surprise, no observable downstream effects of these transcriptomic changes were seen in morphological analyses. It is possible that some form of compensatory mechanism is occurring during development in Plk3-KO mice, but we did not observe upregulation of other Plk family members at the RNA level. At this time, though, despite its robust expression, it appears that Plk3 is dispensable for proper retinal development.

## Materials and Methods

### Ethics Statement

All procedures for the care and housing of mice conform to the U.S. Public Health Service Policy on the Humane Care and Use of Laboratory Animals and were approved by the Institutional Animal Care and Use Committee at Iowa State University.

### Genotyping

Plk3 deficient mice were obtained (Peter Stambrook, University of Cincinnati College of Medicine) and genotyped as described [23]. Plk3 knockouts were defined by the presence of the KO band (F: 5’-AAACCACCTGTGTTGGTGATGTGC-3’; R: 5’-AGCTAGCTTGGCTGGACGTAAACT-3’) whereas wildtype littermates were identified by the presence of a WT band (F: 5’-TTTCCTGGAGCTCTGTAGCCGAAA-3’; R: ACACCCATCTGTGCCATACACTCA-3’). All four primers were used in the same reaction according to the same program: 3 min at 95°C; then 37 cycles of 30s at 95°C, 10s at 60°C, 1 min at 72°C; followed by 7 min at 72°C. The products were separated on a 2% gel.

### Antibody stains

#### Whole-mount Immunohistochemistry

Immediately upon euthanasia, mouse eyes were placed in 4% paraformaldehyde (PFA) in phosphate-buffered saline (PBS) overnight (O/N) at 4°C. After three 15 minute washes in PBS, the retinas were dissected and processed as in [28]. Briefly the retinas were first equilibrated at 4°C in increasing concentrations of sucrose (10%, 20%, 30% sucrose (w/v) in PBS for 30 minutes. After the retinas sank to the bottom of the tube of 30% sucrose/PBS, they were snap-frozen on dry ice and subjected to three freeze/thaw cycles. Retinas could be stored at -80°C in the 30% sucrose/PBS solution. When ready to proceed with the immunostaining, the retinas were rinsed 3 times in PBS for 30 minutes and incubated for 2 hours at room temperature (RT) in blocking solution [3% goat serum/1% bovine serum albumin (BSA)/0.1% Triton-X100/0.02% sodium dodecyl sulfate (SDS) in PBS]. Retinas were then incubated in the different primary antibodies in blocking solution O/N at 4°C on a rocking platform. The retinas were rinsed 3 times in PBS at RT for 30 minutes each and then secondary antibody plus DAPI (10 μg/ml) in blocking solution was added and further incubated O/N at 4°C on a rocking platform. Retinas were rinsed 3 times in PBS at RT for 30 minutes and then flattened between two coverslips for confocal imaging on a Leica SP5 X MP confocal microscope. To quantify each antibody staining, four fields were counted from each retina, two from the nasal side and two from the temporal side.

#### Section Antibody Staining

Immediately upon euthanasia, eyes were placed in 4% (PFA) in PBS O/N at 4°C. After three 15 minute washes in PBS, retinas were isolated and rocked in a solution of 30% sucrose in PBS until they sank (time dependent on mouse age). OCT solution (Tissue-Tek) was added to a concentration of 50% OCT/ 50% sucrose in PBS and retinas were rocked until the solution equilibrated. Upon equilibration, retinas were frozen in blocks at -80°C until cryosectioning. Retinas were sectioned at 20μm and sections placed onto Superfrost Plus microscope slides (Fisher Scientific). Slides were blocked for 30 minutes in PBS-Triton (1% BSA, 0.01% Triton X-100, 0.004% SDS) and then placed in primary antibody, diluted according to manufacturer’s instructions in PBS-Triton, O/N at 4°C. Slides were washed in PBS-Triton three times for 15 minutes at RT. Secondary antibodies were diluted according to manufacturer’s instructions in PBS-Triton and applied either for 2–4 hours at RT or O/N at 4°C. After this incubation, slides were again washed three times for 15 minutes at RT, and mounted with Fluoromount-G (Southern Biotech).

Primary antibodies used were anti-Calbindin28K [Calb28k] [29] (1:2000; Swant, Switzerland), anti-Calretinin [Calr] [29] (1:1000; Millipore), anti-Chx10 (1:1000; [30], anti-Hnf6 [31] (1:200; Santa Cruz Biotechnology) anti-Glutamine Synthetase (1:10,000; Sigma), anti-Rhodopsin [Rho4d2] (1:100; [32]), anti-Choline Acetyltransferase [Chat] (1:100; Millipore), anti-Protein kinase C alpha [PKCα] (1:10,000; Sigma-Aldrich), anti-Recoverin (1:100), anti-Brn3b (1:100; Santa Cruz Biotechnology), anti-Brn3a (1:500; Chemicon MAB1585), anti-Opn4 (1:1000; Advanced Targeting Systems), anti-Ap2a (1:200; Santa Cruz Biotechnology) and anti-PH3 (1:500; Millipore). The anti-Pax6 (1:50) and anti-Islet1 (1:50) antibodies were obtained from the Developmental Studies Hybridoma Bank (DSHB), developed under the auspices of the NICHD and maintained by the University of Iowa, Department of Biology, Iowa City.

### *In Situ* Hybridization

Sequences (between 650 and 800bp in length) were amplified by PCR from mouse cDNA. Probes were visualized using an anti-digoxigenin (DIG)-AP antibody (Roche) and subsequent exposure using BCIP and NBT [28]. The 3’-targeted Plk3 probe used in this study was from the BMAP collection, accession number AW488956, the sequence for which was confirmed before use by sequencing. A probe was also designed for the center of the gene (F: tgtctcctgcttggtgagtg; R: cccgtagaagttcacctgga) and the 5’ portion (F: ctcatcaccgaccctctcag, R: ttgatgcagcggtatgtctc). Fluorescent dissociated cell *in situ* hybridization was also performed as described [33]. Briefly, one probe was synthesized with DIG-labeled nucleotides and the other probe was labeled with fluorescein. Tyramide amplification (Promega) was performed for 10 minutes for the first probe, followed by inactivation in 0.3% hydrogen peroxide. The second probe was then processed in exactly the same manner as the first probe, but with a second color. Six independent fields were photographed and quantified.

### Microarrays

Microarray hybridization was performed as described previously [28]. Briefly, RNA was isolated from retinas using Tri-reagent (Sigma) according to standard manufacturer’s protocols. 400ng of total RNA was used to generate aRNA, from which 5μg were fragmented using the Ambion MessageAmp^™^ II aRNA Amplification Kit according to manufacturer’s instructions. Samples were hybridized to Affymetrix GeneChip Mouse Genome 430 2.0 arrays at the GeneChip facility at Iowa State University. The data are available at NCBI Gene Expression Omnibus (GEO) number GSE75382. Microarray results were analyzed using the Bioconductor Affy package in R [34]. Mas5 was employed for background adjustment and normalization and all data were log(2) transformed. We limited the differential expression analyses to only those genes whose mean log(2) transformed expression value in either WT or KO retinas exceeded the minimum cutoff of 7 as this level of expression was consistently labeled as present by the Affymetrix algorithm. A two-tailed t-test that resulted in p-values of less than 0.05 indicated significant differential expression. The heatmaps were generated using Genesis software [35].

### qPCR

Retinal RNA was isolated using Tri-reagent (Sigma) according to manufacturer’s instructions. 400ng of RNA was used to generate cDNA using random primers and SuperScript III (Life Technologies) according to standard protocols. SybrGreen MasterMix (ThermoFisher) was used to perform qPCR in a BioRad CFX96 Real Time System with BioRad C1000 Thermal Cycler. The program used for qPCR was: 15 min at 95°C, followed by 40 cycles of 15s at 95°C, 30s at 56°C, and 30s at 72°C. β-Actin was used to normalize each experimental gene, and further analysis was performed as described [36]. The difference in average ΔΔC(t) values and the difference plus and minus the standard error of the difference were computed on the C(t) scale. The base-2 antilogs of these three values were computed to obtain estimates with error bars on the fold change scale. Results were plotted on a logarithmic scale. qPCR primers were designed as follows: Plk3-3’ (F:cctgcttaggttccaactcg; R:taaagctggtccctgattcc), Plk3-5’ (F:ggtatagcctacgcggtcaa; R:tgtcagcatcctcgaaatga), Rp1 (F:cctatgtccactccctccaa; R:ccagcctggaaaccatacat), Tac1 (F:gatgaaggagctgtccaagc; R:cagcatgaaagcagaaccag).

## Results

### Characterization of Plk3 expression in the mouse retina

**Fig 1:**
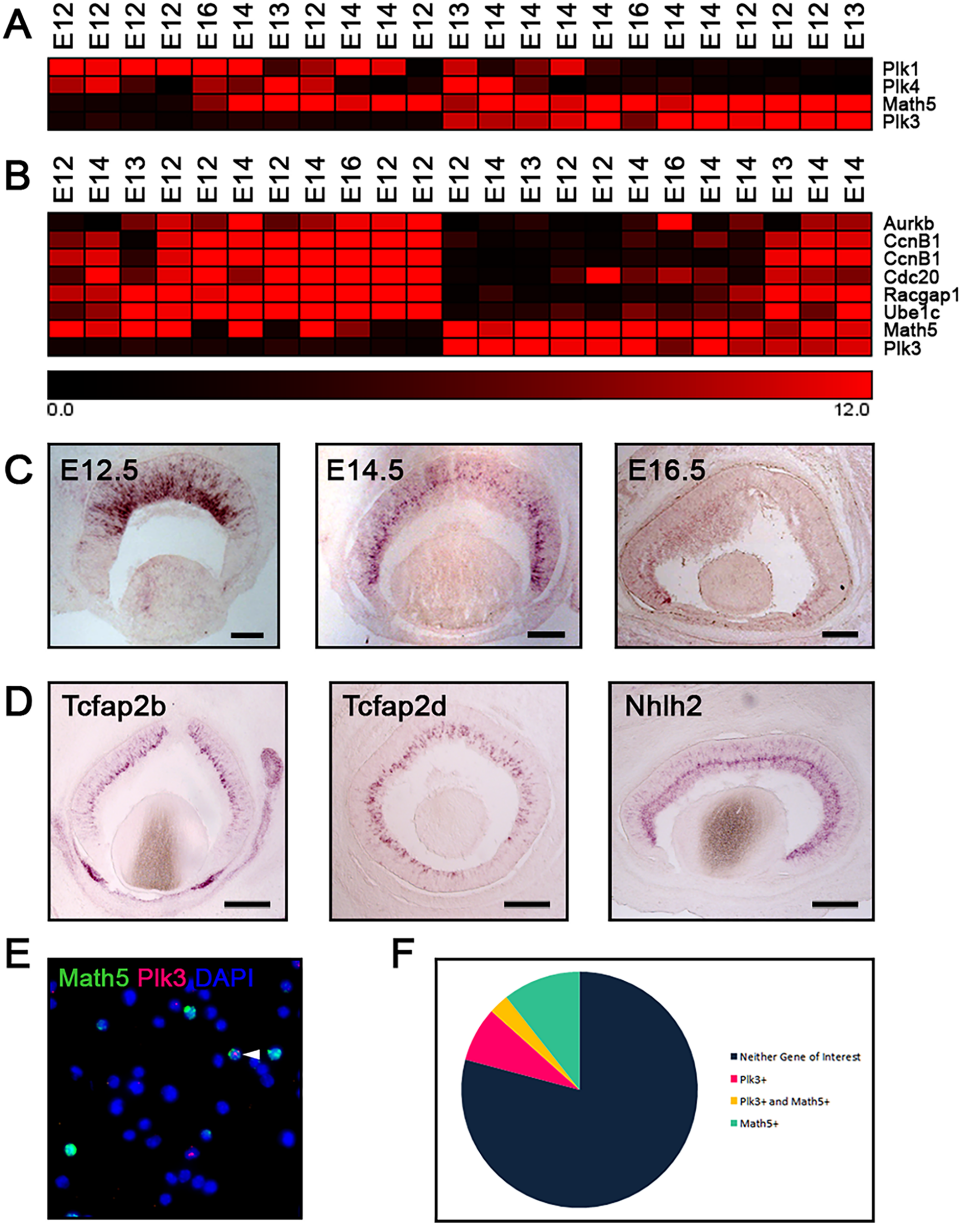
Survey of Plk3 expression in the developing mouse retina. (A) A heatmap showing the expression of Plk family members in Math5-positive cells. The four E12 cells at the left are Math5-negative, but were previously identified as G2/M progenitor cells [2]. These cells are shown as a comparison. In the heatmap, the genes (in rows) expressed in isolated single retinal progenitor cells (in columns) at various stages of development from embryonic day (E)12.5 to E16.5 are shown. Higher levels of microarray signal of a given gene correspond to higher expression levels in a particular single cell and are indicated by the different shades of red (see the scale below [B]), while the absence of expression is indicated with a black square. (B) A heatmap showing G2/M cell cycle marker expression in Math5 and Plk3 expressing single cells. As in (A) the log transformed signal intensities have been scaled according to the intensity of red color, with black indicating the absence of expression signal. The eleven single cells shown on the left are included for comparison and are Plk3-negative cells that were previously identified as in the G2/M phase of the cell cycle [2]. (C) *In situ* hybridization of Plk3 expression in the embryonic mouse retina. (D) *In situ* hybridization showing the expression of Tcfap2b, Tcfap2d, and Nhlh2, markers of developing amacrine interneurons, at E14.5. (E) Double fluorescent *in situ* hybridization of Math5 (green) and Plk3 (red) at E14.5. The results are quantified in (F). All scale bars represent 100 μm.

To attempt to gain insight into the mechanisms of cell fate determination in the retina, the transcriptomes of single retinal progenitors were analyzed. Since Math5 expression correlates with the transition from retinal progenitor cell to differentiating neuron [10, 13, 18], we focused on genes that strongly associated with Math5 expression. Among the genes preferentially expressed in Math5+ retinal progenitors was Plk3 (Fig 1A). In fact, Math5 and Plk3 were among the highest correlated genes in a dataset of embryonic single cells with heterogeneous gene expression [2] (Trimarchi and Cepko, *in preparation*). The polo-like family of kinases have been shown to behave as regulators of cell division in mammalian cells [20, 22, 37], but the specific function of Plk3 *in vivo* is still in question. Since polo-like kinases may have overlapping functions [38], we examined the expression of other Polo-like kinases in our transcriptome dataset of embryonic retinal single cells. It was interesting to note that while other family members, including Plk1 and Plk4, were expressed in subsets of our retinal progenitor cell transcriptomes, the expression of neither Plk3 nor Math5 was strongly correlated with other polo-like kinases (Fig 1A). To determine whether Plk3 expression in the retina correlated with other markers of cell cycle progression, we looked at the expression of G2/M marker genes in Plk3+ and Plk3- cells (Fig 1B). Despite Plk3’s previously described functionality as a cell cycle regulator and the role of these kinases in G2/M progression [20, 22], there was no correlation between Plk3 expression and other markers of G2/M during retinogenesis. In fact, Plk3+ cells were less likely to exhibit markers of G2/M than the Plk3- cells in our set of embryonic retinal progenitor cells. Furthermore, Plk3 did not associate with any cell cycle markers of the G1 or S phase in our hierarchical clustering analysis (data not shown). Plk1 did strongly associate with the G2/M markers demonstrating that our clustering method was in fact robust (data not shown).

We sought to further characterize the expression of Plk3 throughout the course of retinogenesis. *In situ* hybridization (ISH) was performed on frozen retinal sections derived from several different stages of developing mice. At embryonic day (E)12.5, Plk3 mRNA was detected in a subset of cells in the outer neuroblastic layer and most strongly in the inner neuroblastic layer, an area inhabited by newly postmitotic retinal neurons (Fig 1C). By E14.5 the expression of Plk3 mRNA was detected in a line of cells running through the center of the section (Fig 1C). Interestingly, the location of Plk3 expression at E14.5 overlaps with the position of developing amacrine cells (Fig 1C) as other markers of maturing amacrine interneurons have notably similar patterns of expression at this stage in retinal development (Fig 1D). By E16.5, Plk3 expression decreased to undetectable levels in most of the retina, save for the most peripheral region (Fig 1C) and was completely undetectable at postnatal day (P)0 (data not shown). These expression patterns place Plk3 at a time and place to affect the normal development of early-generated retinal cells. Since Math5 and Plk3 were co-expressed in individual retinal cells on microarrays, we wished to explore the specific overlap between larger numbers of Math5+ and Plk3+ cells by labeling them in tandem through dissociated fluorescent *in situ* hybridization (Fig 1E). In dissociated E14.5 retinal cells, 10.2% of cells expressed Plk3, while 13.4% of cells expressed Math5, and 2.8% of all cells showed co-expression of both Plk3 and Math5 (Fig 1F).

### Characterization of Mature and Postnatal Plk3 deficient retinas

Since we observed that Plk3 was expressed in a time and place consistent with the protein having a possible effect on early-generated retinal neurons, we decided to investigate the function of Plk3 in the developing retina. A Plk3-KO mouse had been previously generated, but the retinal development of these mice had not been characterized [23]. We obtained these mice and upon initial characterization noticed that their retinas were degenerated. This was because the mice were partially in a Black Swiss background, which carried a fast-acting allele of retinal degeneration (Pde6b^-/-^, Rd1) [39]. To remove the Rd1 allele from our line, Plk3 heterozygous mice were crossed with wildtype C57Bl/6 mice until all litters were wildtype at the Rd1 locus as assessed by genotyping PCR [40].

**Fig 2:**
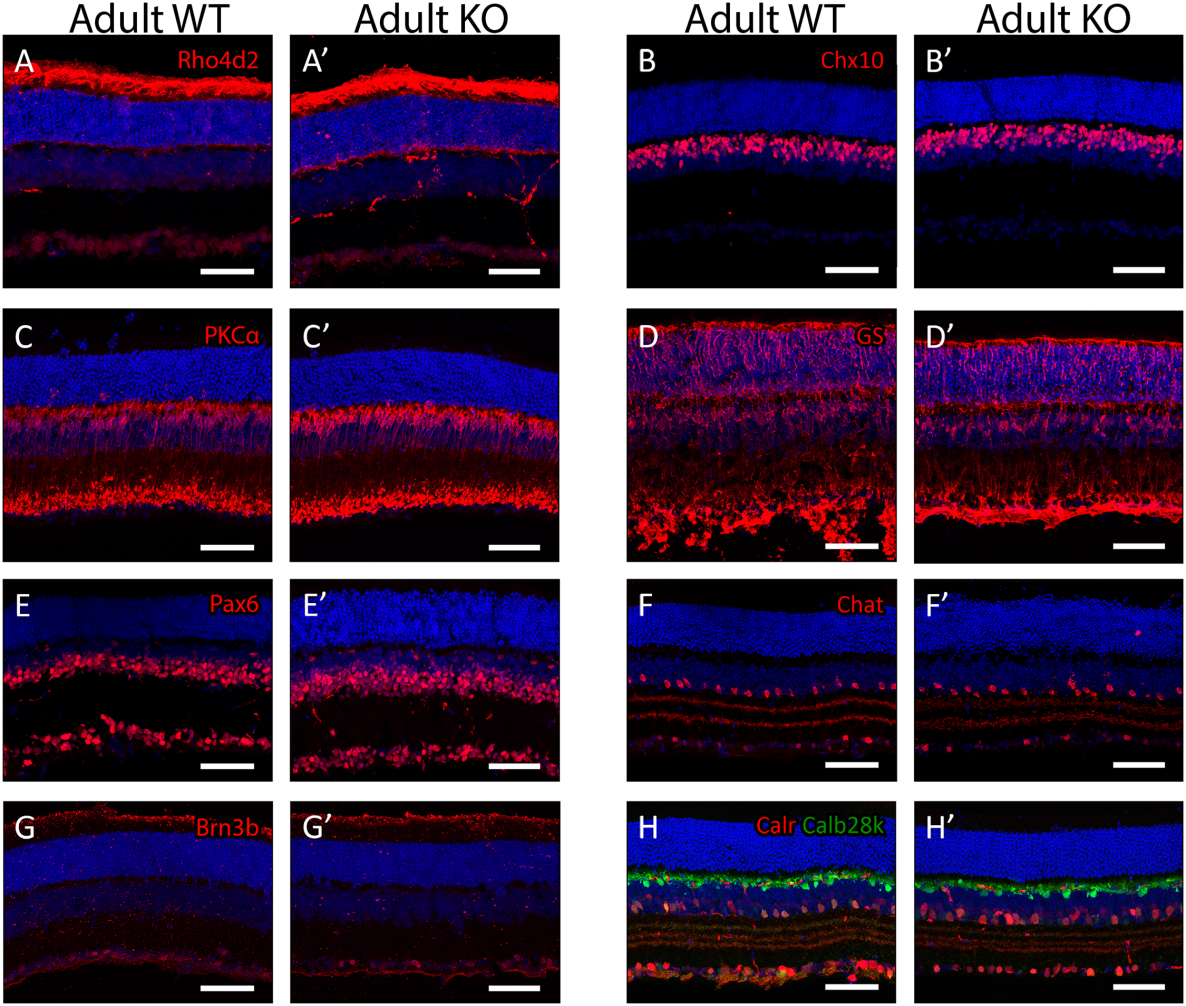
Morphological characterization of adult Plk3-KO retinas. Populations of adult (>P35) retinal cells were identified using antibodies to neuron-specific markers. DAPI, in blue, marks nuclei. Rod photoreceptors (anti-Rhodopsin [Rho4d2], A,A’), bipolar interneurons (anti-Chx10, B,B’; anti-PKC-α, C,C’), Müller glia (anti-Glutamine synthetase [GS] D,D’), amacrine interneurons (anti-Pax6, E,E’; anti-Chat, F,F’), retinal ganglion cells (anti-Brn3b, G,G’), and horizontal, amacrine and ganglion cells (anti-Calretinin [Calr], anti-Calbindin28k [Calb28k], H,H’) are shown. Scale bars represent 100 μm.

To determine whether Plk3 deficiency led to gross disturbances in retinal morphology, we performed antibody stains on adult Plk3-KO retinas and their wildtype (WT) littermates (Fig 2). These surveys of retinal cell populations showed no qualitative differences in populations of rod photoreceptors (anti-Rho4d2, Fig 2A and 2A’), bipolar cells (anti-Chx10, Fig 2B and 2B’; anti-PKC-α, Fig 2C and 2C’), Müller glia (anti-glutamine synthetase, Fig 2D and 2D’), amacrine cells (anti-Pax6, anti-Chat; Fig 2E–2F and 2E’–2F’), retinal ganglion cells (anti-Brn3b, Fig 2G and 2G’), or the combination of horizontal cells and amacrine cells (anti-Calbindin28k, anti-Calretinin; Fig 2H and 2H’) between Plk3-KO and WT mice. Next we wished to assess whether the loss of Plk3 resulted in defects in smaller subsets of mature retinal cells. To accomplish this, *in situ* hybridization was performed (S1 Fig). Cone photoreceptors were marked by Opn1sw (S1A and S1A’ Fig), rod bipolar cells were visualized using a probe against Sebox (S1B and S1B’ Fig) [41], horizontal cells with a Septin4 probe [42] (S1C and S1C’ Fig), and Müller glia were marked with a probe for Vimentin (S1D and S1D’ Fig). Glycinergic amacrine cells were visualized using an Slc6a9 probe (S1E and S1E’ Fig), while GABAergic amacrine cells were observed with a probe to Gad1 (S1F and S1F’ Fig). Again we did not observe any significant and reproducible differences between WT and Plk3-KO littermates for any of these cell types. To assess whether a phenotype would appear over time, we stained 8 month old wildtype and Plk3-KO retinas with an anti-rhodopsin antibody and observed no degeneration of rod photoreceptors in the Plk3 deficient retina (data not shown). Additionally, staining with both anti-Calbindin28k and anti-Calretinin antibodies failed to reveal any deficits in the amacrine or ganglion cell layers or in the IPL itself at this time point (data not shown).

**Fig 3:**
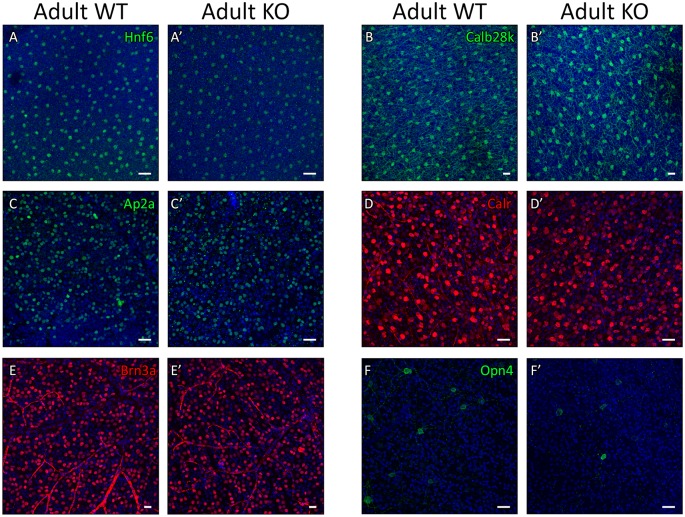
Assessment of cell numbers by flat mount antibody staining. To more quantitatively assess any gains/losses in cell number in the Plk3-KO retina, immunohistochemistry was performed on flat mounted retinas from adult (>P35) wildtype and Plk3-KO littermates. Confocal scans were performed on four different quadrants from each retina and representative quadrant-matched images are shown. DAPI, in blue, marks nuclei. Horizontal cells (anti-Hnf6, A,A’; anti-Calbindin28k [Calb28k], B,B’), amacrine interneurons (anti-Ap2a, C,C’), a combination of amacrine and ganglion cells (anti-Calretinin [Calr], D,D’) and retinal ganglion cells (anti-Brn3a, E,E’; anti-Opn4, F,F’) are shown. Scale bars represent 100 μm.

To more quantitatively assess any potential differences in cell number among the early-generated retinal cell types in the Plk3-KO mice we performed immunohistochemistry on whole retinas. For each antibody utilized, wildtype and Plk3-KO retinas were stained and then four images were acquired from four different quadrants of each retina. We chose to examine the different areas of the retina to assess whether there were any regional differences between wildtype and Plk3-KO retinas that were not captured on the sections. We did not find any significant differences in horizontal cells between wildtype and Plk3-KO retinas using two different antibody markers (anti-Hnf6 [Fig 3A and 3A’; p = .14] and anti-Calbindin28k [Fig 3B and 3B’; p = .73]). To quantify the number of amacrine we used an anti-Ap2a antibody and also found no significant differences in amacrine cells in the different areas of the retina between wildtype and Plk3-KO mice (Fig 3C and 3C’; p = .17). To examine the numbers of retinal ganglion cells, we used three different antibodies: anti-Calretinin, anti-Brn3a and anti-Opn4 (Fig 3D–3F’). Anti-Calretinin antibodies stain ganglion cells and a subset of amacrine cells [29] and this staining did not show any difference between wildtype and Plk3-KO retinas (Fig 3D and 3D’; p = .27). The anti-Brn3a antibody is more specific to ganglion cells, but only stains a subset of them [43]. Again we did not find a significant difference in the numbers of these Brn3a+ ganglion cells between wildtype and Plk3-KO littermates (Fig 3E and 3E’; p = .66). Finally we examined a small subset of ganglion cells, the melanopsin population of intrinsically photosensitive ganglion cells. There are only a small number of cells that stain in any retina with this antibody, but again we did not observe any differences between wildtype and Plk3-KO mice (Fig 3F and 3F’). Taken together, these antibody stains on flat mount retinas indicate that there are no significant differences in the numbers of horizontal, amacrine or ganglion cells in the Plk3-KO retina.

**Fig 4:**
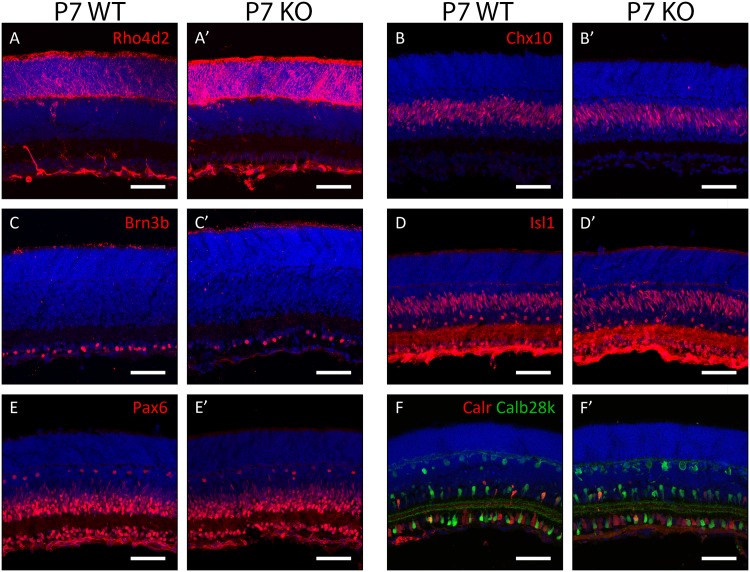
Morphological characterization of postnatal developing Plk3-KO retinas. Populations of retinal cells in postnatal day (P)7 Plk3-KO retinas were compared to WT littermates using cell type-specific markers. Specifically, populations of rod photoreceptors (anti-Rhodopsin [Rho4d2], A,A’), bipolar cells (anti-Chx10, B,B’), retinal ganglion cells (anti-Brn3b, C,C’), bipolar cells, amacrine cells and ganglion cells (anti-Isl1, D, D’), amacrine, horizontal and ganglion cells (anti-Pax6, E,E’; anti-Calretinin [Calr], anti-Calbindin28k [Calb28k], F,F’) were examined. Scale bars indicate 100 μm.

**Fig 5:**
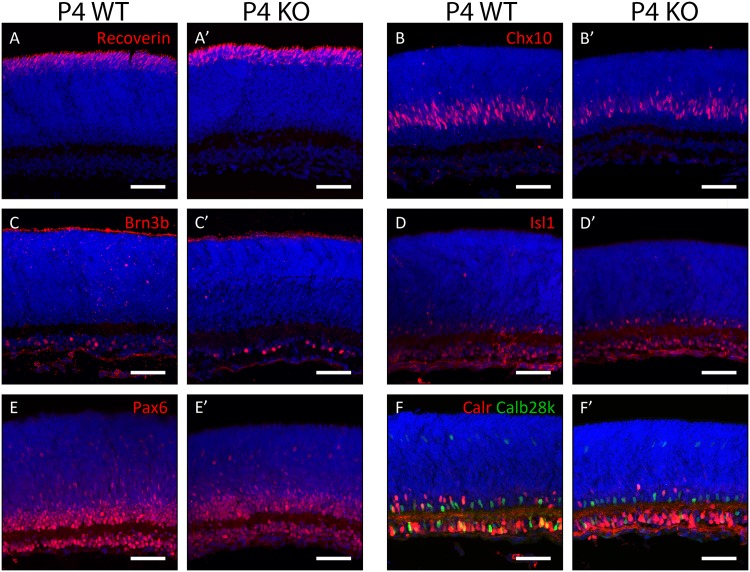
Survey of retinal cell types in P4 Plk3-KO retinas. Populations of retinal cells in P4 Plk3-KO retinas were compared to WT littermates using cell type-specific markers for photoreceptors (anti-Recoverin, A,A’), bipolar cells (anti-Chx10, B,B’), retinal ganglion cells (anti-Brn3b, C,C’), ganglion and amacrine cells (anti-Isl1, D,D’; anti-Pax6, E,E’) and amacrine, horizontal and ganglion cells (anti-Calretinin [Calr], anti-Calbindin28k [Calb28k], F,F’). Scale bars indicate 100 μm.

**Fig 6:**
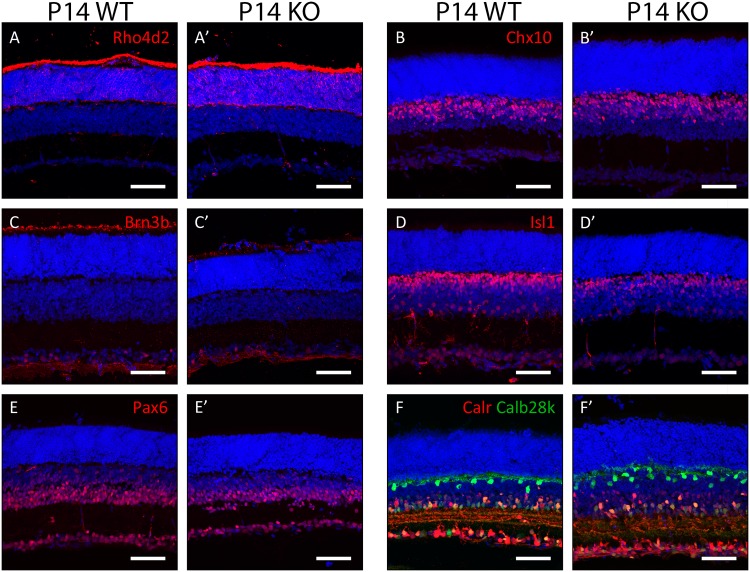
Survey of retinal cell types in P14 Plk3-KO retinas. Populations of retinal cells in P14 Plk3-KO retinas were compared to WT littermates using cell type-specific markers for rod photoreceptors (anti-Rhodopsin [Rho4d2], A,A’), bipolar cells (anti-Chx10, B,B’), retinal ganglion cells (anti-Brn3b, C,C’), ganglion and amacrine cells (anti-Isl1, D,D’; anti-Pax6, E,E’) and amacrine, horizontal and ganglion cells (anti-Calretinin [Calr], anti-Calbindin28k [Calb28k], F,F’). Scale bars indicate 100 μm.

To identify whether any transient developmental phenotypes were present in the Plk3-KO mouse, immunohistochemistry was performed on retinas isolated from earlier stages. Antibody stains at P7 revealed no robust differences between populations of rod photoreceptors (anti-Rho4d2, Fig 4A and 4A’), bipolar cells (anti-Chx10, Fig 4B and 4B’), ganglion cells (anti-Brn3b, Fig 4C and 4C’), amacrine cells (anti-Isl1, anti-Pax6; Fig 4D–4E and 4D’–4E’), and the combination of horizontal cells and amacrine cells (anti-Calbindin28k, anti-Calretinin; Fig 4F and 4F’). To further investigate postnatal development in the Plk3-KO retina, antibody stains were also performed at an earlier (P4, Fig 5) and later (P14, Fig 6) time point. At P4, the populations of maturing photoreceptors (anti-Recoverin, Fig 5A and 5A’), bipolar cells (anti-Chx10, Fig 5B and 5B’), ganglion cells (anti-Brn3b, Fig 5C and 5C’), amacrine interneurons (anti-Isl1, anti-Pax6; Fig 5D–5E and 5D’–5E’), and horizontal cells and amacrines (anti-Calbindin28k, anti-Calretinin; Fig 5F and 5F’) appeared grossly normal between WT and Plk3-KO mice. Similarly, no changes were detected in populations of rod photoreceptors (anti-Rho4d2, Fig 6A and 6A’), bipolar cells (anti-Chx10, Fig 6B and 6B’), ganglion cells (anti-Brn3b, Fig 6C and 6C’), amacrine cells (anti-Isl1, anti-Pax6; Fig 6D–6E and 6D’–6E’), and the combination of horizontal cells and amacrine cells (anti-Calbindin28k, anti-Calretinin; Fig 6F and 6F’).

### Unbiased transcriptomic screening of Plk3 knockout retinas

**Fig 7:**
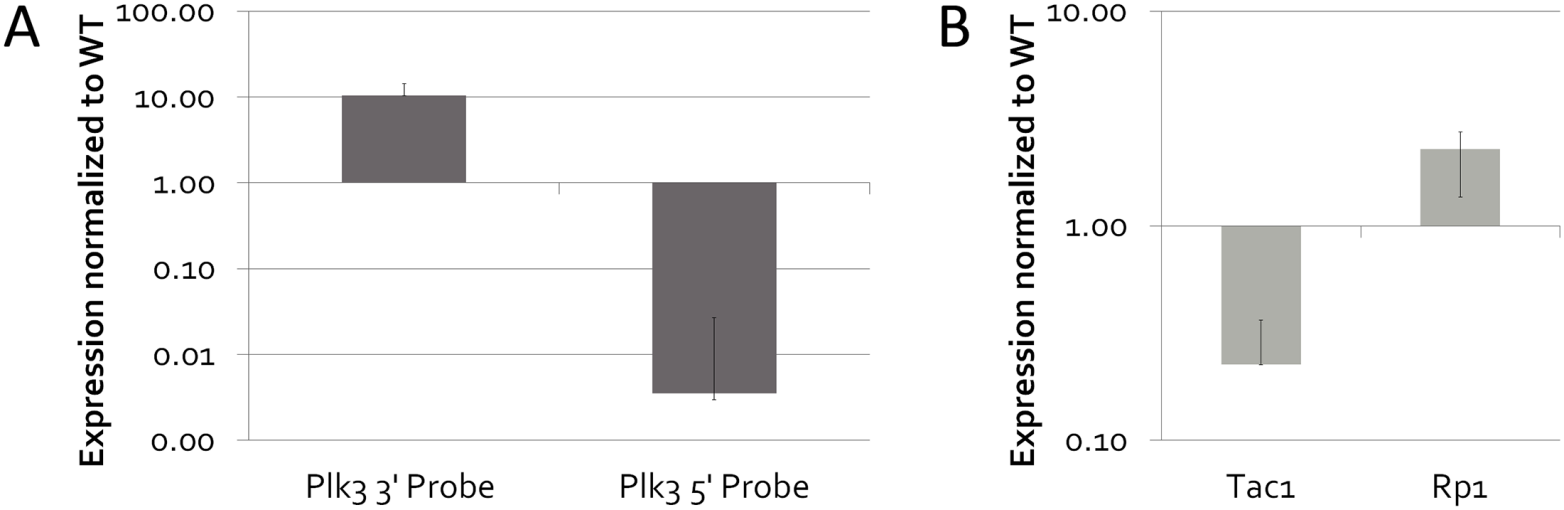
qPCR based examination of gene expression in Plk3-KO retinas. Retinas of Plk3-KO and WT littermates were isolated and hybridized to Affymetrix microarrays at various timepoints (n = 3 for each of four timepoints). Genes with significant differential expression at multiple timepoints were confirmed using qPCR at adult timepoints. (A) An examination of Plk3 expression in the Plk3 deficient mouse. An amplicon at the 3’ end of the gene showed upregulation (p<0.001), while one at the 5’ end of Plk3 was significantly downregulated (p<0.05). (B) Tac1 showed decreased expression (p<0.05), whereas retinitis pigmentosa 1 (Rp1) displayed increased expression (p<0.05).

**Fig 8:**
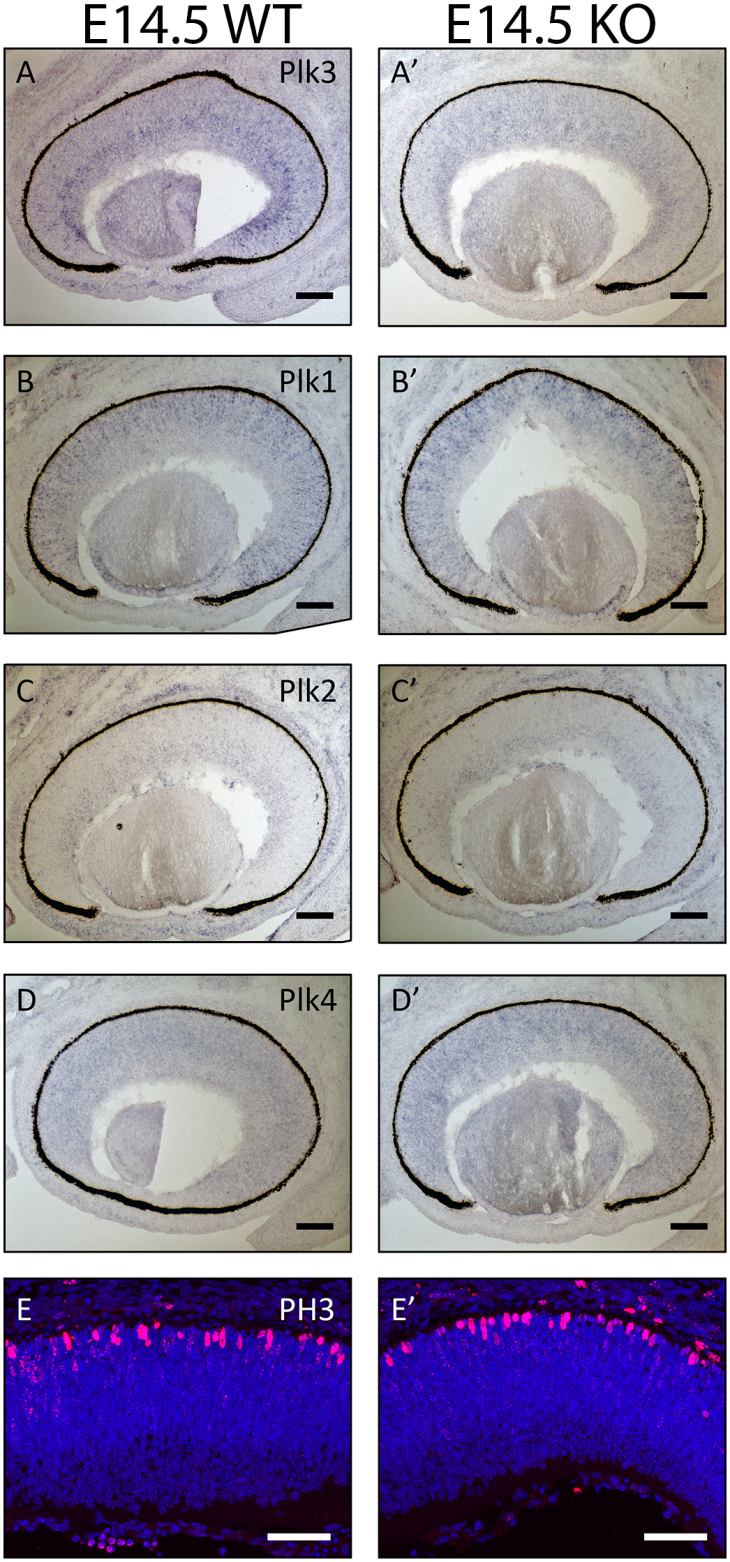
Examination of Plk family member expression patterns and cell cycle at E14.5. The expression patterns of Plk3 (A, A’), Plk1 (B,B’), Plk2 (C,C’), and Plk4 (D,D’) in WT and Plk3-KO retinas were determined using *in situ* hybridization at E14.5. E14.5 developing WT and Plk3-KO retinas were also stained using anti-phospho-histone H3 (PH3) to mark mitotic cells (E,E’).

To better understand the role that Plk3 may play in retinal development, we searched for the presence of more subtle changes in adult retinas that were either wildtype or lacking Plk3. Full transcriptomic analysis of murine retinas not only has the potential to reveal phenotypes that are not immediately apparent upon gross morphological analysis, but can also provide an unbiased determinant of changes in even small neural subpopulations [28]. Therefore, microarray analyses were performed on adult Plk3-KO retinas and their littermates (n = 3 Plk3-KO, n = 3 WT) and on retinas isolated from several time points during development (S1–S4 Tables). Surprisingly, Plk3 itself was consistently among the genes with the highest increase in Plk3-KO mice in our microarray-based expression analysis (S5–S8 Tables). As a similar phenomenon was observed previously for a different knockout mouse [28], the microarray probe for Plk3 was examined and confirmed to contain just the 3’ end of the gene, beyond the extent of the region targeted in the knockout. A qPCR probe designed to the same portion of the gene confirmed significant upregulation of the 3’ end of the gene in the knockout retina (Fig 7A, p<0.01). However, qPCR primers that amplify a region located in the more 5’ functional domain of the Plk3 gene that had been targeted showed extreme downregulation, further confirming the fact that the Plk3 coding region had been removed (Fig 7A, p<0.05). Additionally, to establish that the observed expression was indicative of the entire length of the coding sequence of Plk3, ISH probes to two additional regions of Plk3’s coding sequence were designed. ISH analysis from all three probes confirmed that the expression patterns of each probe spanning the length of Plk3 closely mirrored each other in WT retinas, and displayed a lack of signal in our Plk3-KO retinas (Figs 1C, 8A and 8A’ and data not shown).

To attempt to discern more subtle phenotypes in the Plk3-KO retinas, we first examined the adult microarray results for significantly altered genes in Plk3-KO retinas when compared to wildtype littermates. We observed several photoreceptor-expressed genes (Retinitis pigmentosa1 [Rp1], Castor homolog 1 [Casz1], Retinitis pigmentosa1-like 1 [Rp1l1], Mef2c) that were upregulated, while some markers of Müller glia (Vimentin, Tweety homolog 1, Notch regulated ankyrin repeat protein [Nrarp]) [42], bipolar cells (Sebox, Carbonic Anhydrase 8) [41], and amacrine cells (Nhlh2, Tachykinin 1 [Tac1], Tcfap2c) [42] were decreased when compared to WT littermates (S1 and S5 Tables). To verify these changes by a second method, we used qPCR. Tac1, a marker of subsets of amacrines [44], was confirmed to be downregulated in Plk3-KO by qPCR (p<0.05), whereas retinitis pigmentosa 1 (Rp1), a gene expressed in photoreceptors [45], was significantly upregulated (p<0.05) (Fig 7B). It is surprising that only a small subset of genes in each cell type is altered and this may be why we fail to observe any obvious changes in cell number and cell morphology in the Plk3-KO animals. These results may indicate that while there are some quantitative changes taking place in different cell types in the Plk3-KO, they are not at a high enough level to lead to any overt changes in the cell types themselves.

Since Plk3 is expressed mainly during early development, it was also possible any changes that occur upon its deletion are mainly present during development. In addition, we also wanted to compare the differentially expressed genes from the adult microarrays to the genes that may be changed during postnatal development. Therefore, we isolated retinas from P7 and P0 WT and KO littermates and performed microarray analysis to determine genes with differential expression in retinas that were in different stages of postnatal development (S2 and S3 Tables). Overall, multiple genes were consistently changed between the nine samples studied in all three time points, including Plk3 again, which was among the most upregulated genes in the retina (n = 9 WT, n = 9 Plk3-KO) demonstrating that our transcriptome preparation was highly reproducible (S6 and S7 Tables). Genes related to RNA binding, such as Khdrbs1, were upregulated in the absence of Plk3 (S6 and S7 Tables). Other genes showing significant differential expression included those with as-yet undescribed function in the retina, such as Med8, which was highly downregulated in Plk3-KO retinas (S6 and S7 Tables). Additionally, Cap1, which can play a role in promoting actin cytoskeleton dynamics [46], was highly and consistently upregulated among all postnatal timepoints studied (S5, S6 and S7 Tables). One consistent aspect of all these differentially expressed genes was their presence on chromosome 4 in the mouse, the same chromosome where the Plk3 gene is also located. This may indicate that there is a more chromosomal-wide misregulation of genes present on this chromosome as a result of the nature of the disruption in the Plk3 gene.

A GO enrichment survey was performed using DAVID [47, 48] to determine whether there were any trends in the genes that were significantly differentially expressed at any of the postnatal timepoints (S9 Table). GO analysis showed that genes that were significantly upregulated in Plk3-KO retinas were significantly enriched for terms including phosphorylation and kinase-related genes, positive regulation of transcription and RNA processing, cell differentiation and nuclear export. Enriched GO terms for genes downregulated in the absence of Plk3 included ATP-binding, transcriptional regulation, and cell projection or neurite-related genes. Given that Plk3 is a kinase, some of these classes of genes are consistent with expectations and, therefore, warrant further study.

### Effects of Plk3 loss during embryonic stages of retinogenesis

The expression patterns of Plk3 during the mid-embryonic period of retinal development warranted further investigation into the role of Plk3 during this critical period in cell fate determination. First, since we were concerned about possible compensation from other Plk family members, *in situ* hybridization was performed to compare the expression of Plk3 and its family members at E14.5, the peak of Plk3 expression (Fig 8). While we observed the loss of Plk3 at the mRNA level in sections from Plk3-KO retinas (Fig 8A and 8A’), *in situ* hybridizations for other Plk family members did not appear to change significantly upon the loss of Plk3 in any fashion that would indicate compensation (Fig 8B–8D’). Secondly, we examined any changes in the retinal progenitor cell number by staining mitotic progenitors with anti-phospho-histone H3 antibody (Fig 8E and 8E’) and total progenitor cells with Ki67 (data not shown), but found no significant changes in the number of cycling cells using either antibody.

Plk3 expression is strongest at E14.5 and then begins to decrease by E15.5, and is almost completely gone by E16.5 (Fig 1), by which time the effects of its loss may be apparent in retinal development. Therefore, we performed a full survey of the cell types and transcriptome of E16.5 embryonic retinas to determine whether or not Plk3 loss would lead to subtle or transient changes in retinogenesis. *In situ* hybridization was performed on Plk3-KO mice and their littermates to determine changes in progenitor populations (Chx10, S2A and S2A’ Fig), retinal ganglion cells (Sncg, S2B and S2B’ Fig; Brn3b, S2C and S2C’ Fig), developing photoreceptors (Otx2, S2D and S2D’ Fig) and amacrine interneurons (Ap2a, S2E and S2E’ Fig; Ap2b, S2F and S2F’ Fig). While *in situ* hybridization is not a quantitative assay, we did not observe any discernable differences in these developing retinal populations.

To better understand the changes that may result from Plk3 loss in the mid-embryonic retina, including those that would not be apparent from whole tissue sections, transcriptomic analysis of E16.5 retinas was performed (S4 Table). To minimize biological variation, retinas from 3 Plk3-KO and WT littermates were isolated. Among the differentially-expressed genes were the retinitis pigmentosa GTPase regulator interacting protein (Rgrip1), which was highly upregulated in the absence of Plk3 and axon targeting molecules and receptors such as Sema3f, Sema4d, Epha8, and Ephb3 [49] that were downregulated (S8 Table). GO analysis determined that clusters of genes associated with catabolic processes, cell and neurite projection, and phosphorylation were significantly enriched in WT Plk3 mice compared to their Plk3-null littermates at E16.5 (S9 Table). Conversely, genes significantly overexpressed in Plk3-KO mice disproportionally represented clusters of genes associated with ion binding, chromatin organization and regulation, RNA binding and transcriptional processes (S9 Table). Since we failed to observe any changes in dendritic morphology in adult retinas, the repercussions of these changes to the overall retinal function remain unknown.

## Discussion

To try and gain a better understanding of retinal progenitor cell behavior, we examined the single cell transcriptomes of Math5 expressing cells isolated from embryonic mouse retinas [2]. We found that the expression of Polo-like kinase 3 was highly correlated with the expression of Math5 in our single cell data set. Plk3 is a member of the polo-like family of kinases, members of which have been previously shown to play important roles in cell cycle progression [20, 22, 50, 51]. In particular, Plk3 has been hypothesized to work together with Cdc25a to decrease Cyclin E levels post-transcriptionally as a means of regulating progression from G1 to S phase [22, 50]. Plk3 and its related family member Plk2 have also been implicated in non-cell cycle processes including synaptogenesis and the maintenance of neurite integrity [25, 26]. Despite its potential roles in multiple important cellular processes, Plk3 has not yet been characterized in the retina, either by expression or by function. Therefore, when we found that it was one of the most highly correlated genes with Math5 expression in our single cell data, we sought to examine its expression in embryonic retinas and assess its role during retinal development. Surveys of Plk3’s expression in the embryonic retina revealed that this gene was expressed in patterns reminiscent of Math5 expression, with strong expression in a subset of cells at both E12.5 and E14.5. At this point, the expression of Plk3 deviated from that of Math5. Math5 expression is strong at E16.5, while Plk3 expression is confined to the far periphery of the retina. The expression pattern of Plk3 in the early retina was even more reminiscent of markers of developing amacrine cells such as Tcfap2b, Tcfap2d, and Nhlh2, especially at E14.5. Altogether the expression data for Plk3 pointed to possible phenotypes in early-generated retinal neurons, particularly the amacrine cell population.

To better understand the role played by Plk3 in the neural retina, we obtained a Plk3 deficient mouse [23] and performed a thorough morphological and transcriptomic analysis searching for any differences present between Plk3-KO mice and their WT littermates throughout development. To our surprise, no distinctive reproducible morphological changes were observed in adult Plk3-KO mice when compared with their WT littermates. This lack of differences between the Plk3-KO and its WT littermates was consistent across all stages of development examined, both postnatal and embryonic. Although we were initially surprised by the lack of a discernable phenotype, perhaps we should not have been. Studies of mice deficient for members of the Tcfap and Nhlh families of transcription factors also failed to reveal discernable phenotypes or even subtle phenotypes, possibly resulting from functional redundancy among family members [52, 53]. Tcfap2b, Tcfap2d, and Nhlh2 showed very similar expression patterns as Plk3 during retinal development, which may point to all of these genes being involved in the development of the same small population of cells. Redundancy was one hypothesized reason for why phenotypes were not observed in these other knockout mice. The Plk family members exhibit high levels of conservation that could indicate overlapping functionality [21] and all of the family except Plk2 were detected in the developing retina. While we noted no significant changes in the mRNA expression levels of other Plk family members upon loss of Plk3, this observation does not preclude that even small amounts of enzymatic activity from other family members could compensate for the loss of Plk3.

There have been multiple examples of non-phenotypic mice that exhibit genetic robustness; that is, the knocked-out gene carries no observable phenotypic weight as a result of adaptation or as an inherent feature of a genetic pathway with built-in failsafe mechanisms [54]. An additional concern when considering the phenotype of a knockout mouse is the variability in background strain and mechanism of generation of the knockout, including the nature of deleted exons. For instance, while the mouse used in this study was generated with a knockout of exons 1–6, it was not shown to contribute to tumorigenesis [23]. However, another Plk3-KO mouse generated by removal of exons 1–8 did show increased tumor formation in mice of advanced age [55]. Both deletions of the Plk3 gene included the active sites for the protein and should have resulted in a non-functional Plk3 protein (if any protein was produced). However, the possibility remains that the background of the mice or the methods employed to identify a phenotype contributed to either study’s findings as well as those of the current investigation. Perhaps with better markers for different retinal cell subsets, especially amacrine cells, it will be possible in the future, to discern a subtle morphological phenotype, such as the loss of a small subtype of amacrines, in the Plk3 deficient retinas.

Even though we did not observe any reproducible morphological alterations in the Plk3-KO animals, we did observe transcriptomic changes through microarray experiments performed at various time points. It is unclear what these changes mean, as Plk3 is not a transcription factor, but rather a kinase enzyme. However, the alterations in gene expression were observed across multiple biological replicates and were additionally confirmed by qPCR. These changes, especially to the gene Tac1, might be indicative of subtle phenotypes in small populations of amacrine cells and warrant further investigation. Additionally, these array experiments revealed genes that were changed across multiple different timepoints. Upon further examination it was noticed that these genes happened to reside on the same chromosome (chromosome 4) as Plk3. It is entirely possible that the changes in expression of these genes are due to the nature of the Plk3 deletion. Surprisingly, these genes do not all lie in close proximity to Plk3 itself so the nature of how the deletion affected their expression is unclear. Taken together though, these observations serve as a potential caution for future studies assessing transcriptomic changes in knockout animals of this nature.

## Supporting Information

S1 Fig *In situ* hybridization of adult Plk3-KO retinas. *In situ* hybridization was employed to determine the effects of Plk3-deficiency on adult retinal cells. Probes staining short-wave cones (Opn1sw, A, A’), rod bipolar cells (Sebox, B, B’), horizontal cells (Septin 4, C, C’), Müller glia (Vimentin, D, D’), glycinergic amacrine cells (Slc6a9, E, E’) or GABAergic amacrine cells (Gad1, D,D’) were utilized. Scale bars represent 100 μm.
(TIF)

S2 Fig Examination of expression patterns in E16.5 Plk3 deficient retinas. *In situ* hybridization was employed to determine any effects of Plk3-deficiency present at E16.5. The probes used were Chx10 [A,A’] (progenitor cells), Synuclein gamma [Sncg](RGCs) [B,B’], Brn3b (RGCs) [C,C’], Otx2 (developing photoreceptors) [D,D’], Ap2a (ACs) [E,E’], and Ap2b (ACs) [F,F’]. Scale bars represent 100 μm.
(TIF)

S1 Table Adult array data. Affymetrix array data for n = 3 adult Plk3 deficient retinas and n = 3 corresponding wildtype littermate control retinas. The data was extracted from the cel files using the Affy R package developed by Bioconductor [49]. After background adjustment and normalization using Mas5, the data were log(2) transformed.
(XLSX)

S2 Table P7 array data. Affymetrix array data for n = 3 P7 Plk3 deficient retinas and n = 3 corresponding wildtype littermate control retinas. The data was extracted from the cel files using the Affy R package developed by Bioconductor [49]. After background adjustment and normalization using Mas5, the data were log(2) transformed.
(XLSX)

S3 Table P0 array data. Affymetrix array data for n = 3 P0 Plk3 deficient retinas and n = 3 corresponding wildtype littermate control retinas. The data was extracted from the cel files using the Affy R package developed by Bioconductor [49]. After background adjustment and normalization using Mas5, the data were log(2) transformed.
(XLSX)

S4 Table E16.5 array data. Affymetrix array data for n = 3 E16.5 Plk3 deficient retinas and n = 3 corresponding wildtype littermate control retinas. The data was extracted from the cel files using the Affy R package developed by Bioconductor [49]. After background adjustment and normalization using Mas5, the data were log(2) transformed.
(XLSX)

S5 Table Differentially expressed genes from adult WT and Plk3-KO retinas. To be considered for differential expression analysis, the log(2) transformed mean of either n = 3 WT or n = 3 KO expression values must have exceeded 7 to indicate overall expression in either genotype. A two-tailed t-test that resulted in p-values of less than 0.05 was used to indicate significant differential expression.
(XLSX)

S6 Table Differentially expressed genes from P7 WT and Plk3-KO retinas. To be considered for differential expression analysis, the log(2) transformed mean of either n = 3 WT or n = 3 KO expression values must have exceeded 7 to indicate overall expression in either genotype. A two-tailed t-test that resulted in p-values of less than 0.05 was used to indicate significant differential expression.
(XLSX)

S7 Table Differentially expressed genes from P0 WT and Plk3-KO retinas. To be considered for differential expression analysis, the log(2) transformed mean of either n = 3 WT or n = 3 KO expression values must have exceeded 7 to indicate overall expression in either genotype. A two-tailed t-test that resulted in p-values of less than 0.05 was used to indicate significant differential expression.
(XLSX)

S8 Table Differentially expressed genes from E16.5 WT and Plk3-KO retinas. To be considered for differential expression analysis, the log(2) transformed mean of either n = 3 WT or n = 3 KO expression values must have exceeded 7 to indicate overall expression in either genotype. A two-tailed t-test that resulted in p-values of less than 0.05 was used to indicate significant differential expression.
(XLSX)

S9 Table Gene ontology (GO) term enrichment for upregulated genes in the WT and Plk3-KO retinas at each of the stages profiled. GO term enrichment was performed with DAVID (http://david.abcc.ncifcrf.gov/) using default parameters. The p-values are reported as computed by DAVID.
(XLSX)
