# Supplementary figures and images for: Polo-Like Kinase 3 Appears Dispensable for Normal Retinal Development Despite Robust Embryonic Expression
Source: PLoS One. 2016 Mar 7;11(3):e0150878. doi: 10.1371/journal.pone.0150878 (PMC4780821; doi:10.1371/journal.pone.0150878)

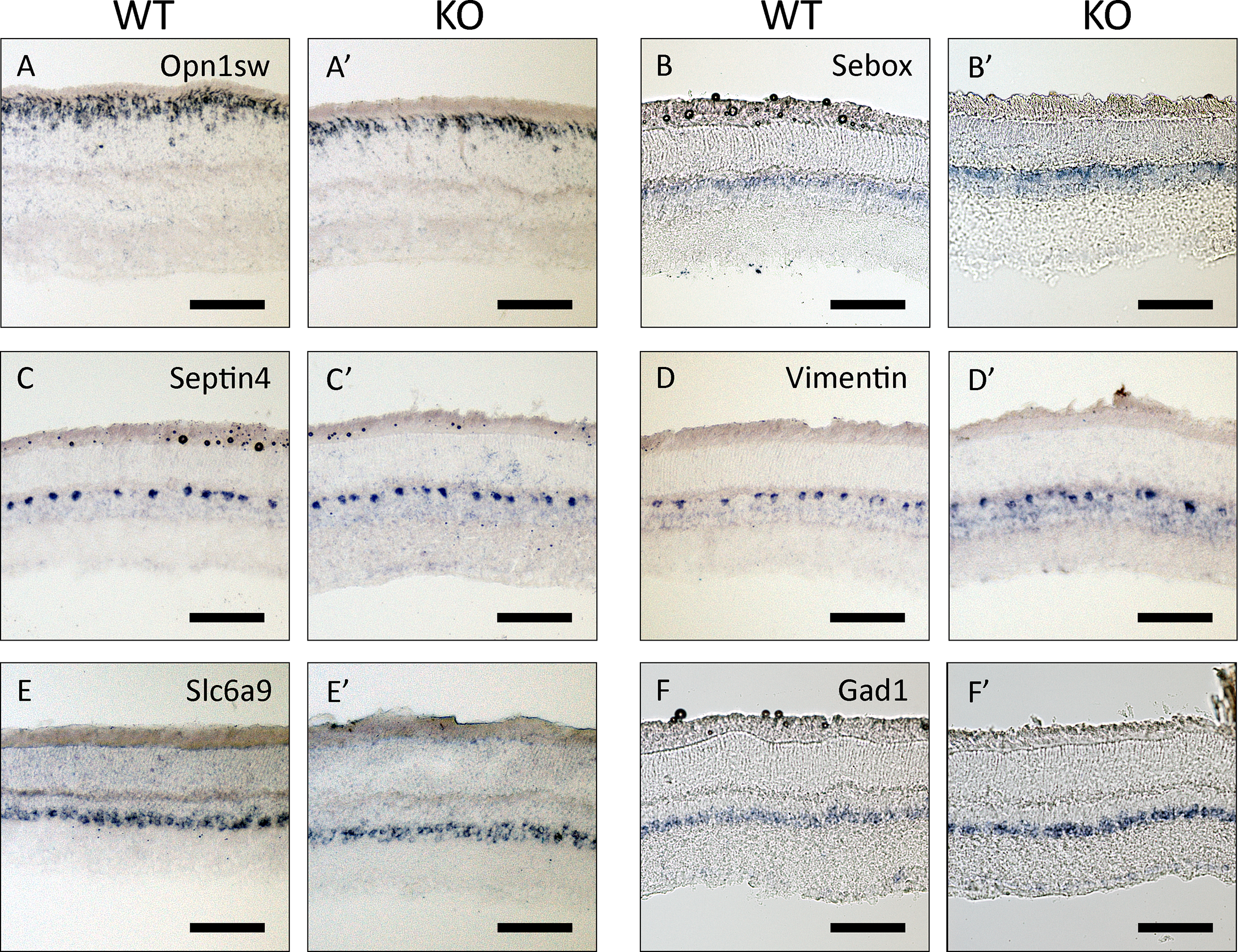

Supplement: S1 Fig — In situ hybridization was employed to determine the effects of Plk3-deficiency on adult retinal cells. Probes staining short-wave cones (Opn1sw, A, A’), rod bipolar cells (Sebox, B, B’), horizontal cells (Septin 4, C, C’), Müller glia (Vimentin, D, D’), glycinergic amacrine cells (Slc6a9, E, E’) or GABAergic amacrine cells (Gad1, D,D’) were utilized. Scale bars represent 100 μm. (TIF) [file pone.0150878.s001.tif]

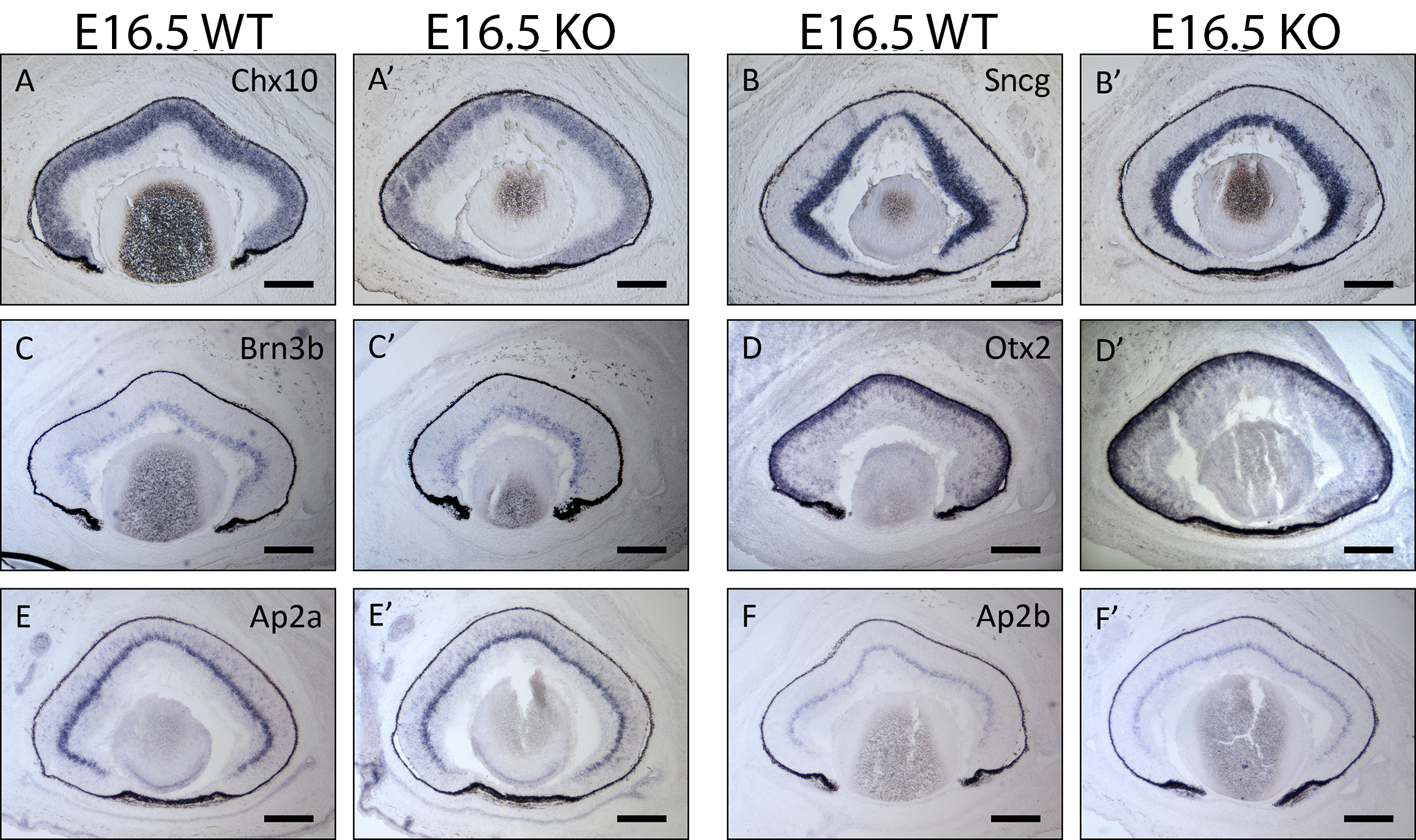

Supplement: S2 Fig — In situ hybridization was employed to determine any effects of Plk3-deficiency present at E16.5. The probes used were Chx10 [A,A’] (progenitor cells), Synuclein gamma [Sncg](RGCs) [B,B’], Brn3b (RGCs) [C,C’], Otx2 (developing photoreceptors) [D,D’], Ap2a (ACs) [E,E’], and Ap2b (ACs) [F,F’]. Scale bars represent 100 μm. (TIF) [file pone.0150878.s002.tif]
